# Relationship between Microstructure and Corrosion Behavior of Martensitic High Nitrogen Stainless Steel 30Cr15Mo1N at Different Austenitizing Temperatures

**DOI:** 10.3390/ma10080861

**Published:** 2017-07-27

**Authors:** Zhouhua Jiang, Hao Feng, Huabing Li, Hongchun Zhu, Shucai Zhang, Binbin Zhang, Yu Han, Tao Zhang, Dake Xu

**Affiliations:** 1 School of Metallurgy, Northeastern University, Shenyang 110819, China; jiangzh@smm.neu.edu.cn (Z.J.); fenghao241@163.com (H.F.); zhc803813@163.com (H.Z.); zscdbdx@163.com (S.Z.); binbin_z12@163.com (B.Z.); han_yu8023@163.com (Y.H.); 2 School of Materials Science and Engineering, Northeastern University, Shenyang 110819, China; zhangtao@mail.neu.edu.cn (T.Z.); xudake@imr.ac.cn (D.X.)

**Keywords:** martensitic high nitrogen stainless steel, austenitizing temperature, microstructure, pit initiation, passive film, pit growth

## Abstract

The relationship between microstructure and corrosion behavior of martensitic high nitrogen stainless steel 30Cr15Mo1N at different austenitizing temperatures was investigated by microscopy observation, electrochemical measurement, X-ray photoelectron spectroscopy analysis and immersion testing. The results indicated that finer Cr-rich M_2_N dispersed more homogeneously than coarse M_23_C_6_, and the fractions of M_23_C_6_ and M_2_N both decreased with increasing austenitizing temperature. The Cr-depleted zone around M_23_C_6_ was wider and its minimum Cr concentration was lower than M_2_N. The metastable pits initiated preferentially around coarse M_23_C_6_ which induced severer Cr-depletion, and the pit growth followed the power law. The increasing of austenitizing temperature induced fewer metastable pit initiation sites, more uniform element distribution and higher contents of Cr, Mo and N in the matrix. In addition, the passive film thickened and Cr_2_O_3_, Cr^3+^ and CrN enriched with increasing austenitizing temperature, which enhanced the stability of the passive film and repassivation ability of pits. Therefore, as austenitizing temperature increased, the metastable and stable pitting potentials increased and pit growth rate decreased, revealing less susceptible metastable pit initiation, larger repassivation tendency and higher corrosion resistance. The determining factor of pitting potentials could be divided into three stages: dissolution of M_23_C_6_ (below 1000 °C), dissolution of M_2_N (from 1000 to 1050 °C) and existence of a few undissolved precipitates and non-metallic inclusions (above 1050 °C).

## 1. Introduction

Corrosion is diagnosed as one of the main factors for premature bearing failures in many aviation applications, particularly in aircraft engines and helicopters [1,2]. The conventional high carbon martensitic stainless steels, such as 440C and BG42, possess some corrosion resistance. However, the existence of coarse Cr-rich eutectic carbides (M_7_C_3_) deteriorates their fatigue capabilities and corrosion resistance [2]. As an important alloying element, nitrogen can significantly improve the corrosion resistance and mechanical properties of stainless steels, and has been widely used in austenitic and duplex stainless steels [3,4,5,6]. Various mechanisms, including theories of ammonia production [7], surface enrichment [8], anodic segregation [9] and salt film formation [10], have been proposed to explain the effect of nitrogen on corrosion resistance of nitrogen-containing steels. For martensitic stainless steels (MSSs), nitrogen in solid solution could also enhance their corrosion resistance [11,12]. Besides, nitrogen is beneficial to improve the hardenability and avoid the segregation of eutectic carbides in MSSs [2]. The partial substitution of nitrogen for carbon could also increase the thermodynamic stability of solid solution, toughness and plasticity of MSSs [13].

However, due to the low nitrogen solubility (normally less than 0.08 wt %) in martensitic steels at atmospheric pressure [11,14], it is difficult to obtain MSSs with high nitrogen content by traditional methods, such as electric arc furnace (EAF) melting, vacuum induction melting (VIM) and argon oxygen decarburization (AOD) refining. Therefore, few studies about martensitic high nitrogen stainless steels were reported [12,15,16,17]. In recent years, with the development of pressure metallurgy, series of MSSs with high nitrogen content (0.3–0.5 wt %), such as CRONIDUR steels, have been invented using pressurized electroslag remelting (PESR) [11]. For example, CRONIDUR 30 has been used successfully in demanding applications, such as bearings for aviation turbine and cryogenic rocket turbopumps [18].

It is well known that austenitizing and tempering strongly influence the microstructure and properties of MSSs [19]. In general, the austenitizing temperature determines the amount and distribution of undissolved carbides and retained austenite [20], and has an important influence on the mechanical properties and corrosion resistance [21]. Meanwhile, decarburization, grain coarsening and the formation of δ-ferrite should be avoided in selecting a suitable austenitizing temperature [19,22]. Several studies have been carried out about the effect of austenitizing treatment on the microstructure and corrosion resistance of nitrogen-free or low nitrogen martensitic stainless steels [20,21,23]. The results indicated that the reduction of undissolved Cr-rich carbides and the improvement in homogeneity of Cr distribution with increasing austenitizing temperature improved the corrosion resistance of steels. The existence of Cr-depleted zones in matrix adjacent to Cr-rich precipitates acted as preferential sites for metastable pits. In addition, Lu et al. [20,23] pointed out that the increased austenitizing temperature contributed to forming more protective and well-structural passive films on 13 wt % Cr-type MSSs. However, few studies can be found about the influence of austenitizing temperature on microstructure, composition of passive film, pit initiation and propagation of martensitic high nitrogen stainless steels.

Martensitic high nitrogen stainless steel 30Cr15Mo1N with 0.44 wt % nitrogen has been developed successfully in Northeastern University using pressure metallurgy method. The microstructure, mechanical and corrosion properties of friction stir welding 30Cr15Mo1N were investigated in our previous study, and the results revealed that the weldment exhibited lower hardness and better corrosion resistance [24]. The present work aims to reveal the relationship between microstructure and corrosion behavior of 30Cr15Mo1N at different austenitizing temperatures, and promote the development and application of martensitic high nitrogen stainless steels. The investigation of characterization of passive film, pitting initiation and propagation, etc. of such steel could afford a broad overall view on pitting corrosion, and is beneficial for understanding the corrosion mechanism of martensitic high nitrogen stainless steels. 

## 2. Materials and Methods

### 2.1. Material and Heat Treatment

**Figure 1:**
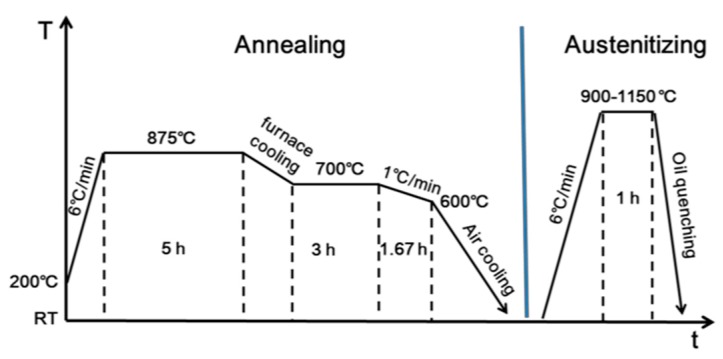
Schematic of heat treatment procedure.

The experimental martensitic high nitrogen stainless steel 30Cr15Mo1N was melted in a 25 kg pressurized induction furnace (Jinzhou Yuanteng Electric Furnace Technology Co., Ltd., Jinzhou, China). During the smelting process, the vacuum carbon-deoxidization together with adding nickel-magnesium (Ni-Mg) master alloy and metallic cerium was used for deoxidization and desulfurization. Then, nitrogen was introduced to the steel via the reaction, N_2_ (g) → 2[N], at the gas-melt interface under nitrogen pressure of 0.4 MPa. Finally, the molten steel was poured into the ingot mold and solidified under nitrogen pressure of 1.5 MPa to avoid the formation of nitrogen bubbles and porosity. The chemical composition of the present material is shown in Table 1. Firstly, the ingot was diffusion annealed at 1270 °C for 10 h, then hot forged into 110 mm × 30 mm plate in the temperature range of 1050–1150 °C. Afterwards, the plate was annealed at 875 °C for 5 h, then furnace cooled to 700 °C and kept for another 3 h, followed by cooling to 600 °C in furnace at the speed of 1 °C/min, finally air-cooled to room temperature (Figure 1). The plate was cut into several parts, then austenitized at 900 °C, 950 °C, 1000 °C, 1020 °C, 1050 °C, 1100 °C and 1150 °C, respectively, for 1 h followed by oil quenching.

### 2.2. Thermodynamic Calculations

The variation of phase fractions and composition with temperature at equilibrium were generated using Thermo-Calc with TCFE7 database (Thermo-Calc Software AB, Solna, Sweden).

### 2.3. Microstructure Characterizations

To reveal the phase constitution, the specimens for X-ray diffraction (XRD) analyses with the dimensions of 12 mm × 10 mm × 6 mm were ground with SiC paper to 2000-grit and mechanically polished with 2.5 μm diamond paste, then electropolished at 20 V in the electrolyte composed of 25 vol % perchloric acid and 75 vol % ethanol. The XRD tests were carried out on an X-ray diffractometer (D/Max-2400, Rigaku, Tokyo, Japan) with Cu Kα radiation under 50 kV, 180 mA and 2θ ranging from 40° to 101° at a scanning speed of 2°/min. The volume fraction of retained austenite was determined by Equation (1) [25]:

(1) $$V_{A}=\frac{1.4I_{\gamma }}{I_{\alpha }+1.4I_{\gamma }}$$

where *I*_γ_ and *I*_α_ are the integrated intensities of (111)_γ_ and (110)_α_, respectively.

To observe the influence of austenitizing temperature on precipitation distribution, specimens with dimensions of 12 mm × 10 mm × 6 mm were ground with SiC paper to 2000-grit and mechanically polished with 2.5 μm diamond paste, then etched by Vilella’s reagent (consisting of 1 g picric acid, 10 mL hydrochloric acid and 100 mL ethanol). Microstructure and element distribution were performed using field emission scanning electron microscope (FE-SEM, Ultra Plus, Carl Zeiss, Oberkochen, Germany) and electron probe microanalyzer (EPMA, JXA-8530F, JEOL, Tokyo, Japan) equipped with wavelength dispersive spectrometer (WDS). The FE-SEM and EPMA were operated with an accelerating voltage of 15.0 kV, and the probe current of EPMA was 5.0 × 10^−^^8^ A. The area percentage of precipitates was quantified using Image-Pro Plus software Version 6.0 (Media Cybernetics Inc., Rockville, MD, USA). The microstructure and lattice information of the precipitates were investigated by transmission electron microscopy (TEM, JEM-2100F, JEOL, Tokyo, Japan) at 200 kV, and the Cr concentration at the precipitate–matrix interface was analyzed using energy dispersive spectrometer (EDS) attached to TEM. For TEM foil preparation, thin disks with diameter of 3 mm were cut from the specimen austenitized at 900 °C and twin-jet polished using the electrolyte composed of 8 vol % perchloric acid and 92 vol % ethanol at −20 °C.

### 2.4. Electrochemical Measurement

The specimens for electrochemical measurements were mounted in epoxy resin with an exposed area of 10 mm × 10 mm and abraded with SiC paper to 2000-grit. The test medium was 3.5 wt % NaCl solution at 30 ± 0.5 °C prepared with analytically pure NaCl and deionized water. The potentiodynamic polarization measurement was performed using a Gamry Reference 600 potentiostat (Gamry, Warminster, PA, USA) with a three-electrode system, consisting of a platinum counter electrode, a saturated calomel electrode (SCE) reference electrode and the specimen as the working electrode. Prior to the measurement, the working electrode was polarized at −1.0 V_SCE_ for 5 min to eliminate the surface oxide layer formed in the air, then stabilized at open circuit potential (OCP) for 30 min. After that, potentiodynamic polarization was performed from −0.3 V below OCP toward the positive direction at a scan rate of 0.333 mV/s, and test was terminated when current density reached 1 mA/cm^2^. 

### 2.5. Passive Film Analysis

The specimens for passive film growth were mechanically abraded and polished. After removing the surface oxide layer by cathodic polarization, the passivation potential, which was chosen based on the potentiodynamic polarization curves, was applied for 1 h to promote the growth of passive film. The passive film analysis was then performed using X-ray photoelectron spectrometer (XPS, ESCALAB 250, Thermo Scientific, Waltham, MA, USA) with Al Kα (1486.6 eV) X-ray source. The binding energies were calibrated relative to C 1s peak at 284.6 eV. The argon ions sputtering (base pressure: 1.33 × 10^−5^ Pa, energy: 2 kV, current: 2.0 μA/cm^2^) over an area of 2 mm × 2 mm were performed for 10 s to clear the contamination layer. The curve fitting was performed via XPSPEAK 4.1 software by referencing to a database [26].

### 2.6. Immersion Tests

The pit growth kinetics was evaluated using immersion testing and metallographic examination. After being abraded with 2000-grit SiC paper, the specimens austenitized at 900 °C, 1000 °C and 1100 °C were immersed in 6 wt % FeCl_3_ solution at 50 ± 0.5 °C according to ASTM G48-11 (Method A) [27]. After being immersed for 3 h, 6 h, 12 h and 24 h, the specimens were taken out of the solution, cleaned using deionized water and dried using cold air. Then the specimens were cleaned in accordance with ISO 8407-2009 [28] to remove the corrosion products. Finally, the variation of pit depth was estimated using laser scanning confocal microscope (LSCM, LEXT OLS4100, Olympus, Tokyo, Japan).

## 3. Results

### 3.1. Thermodynamic Calculations

**Figure 2:**
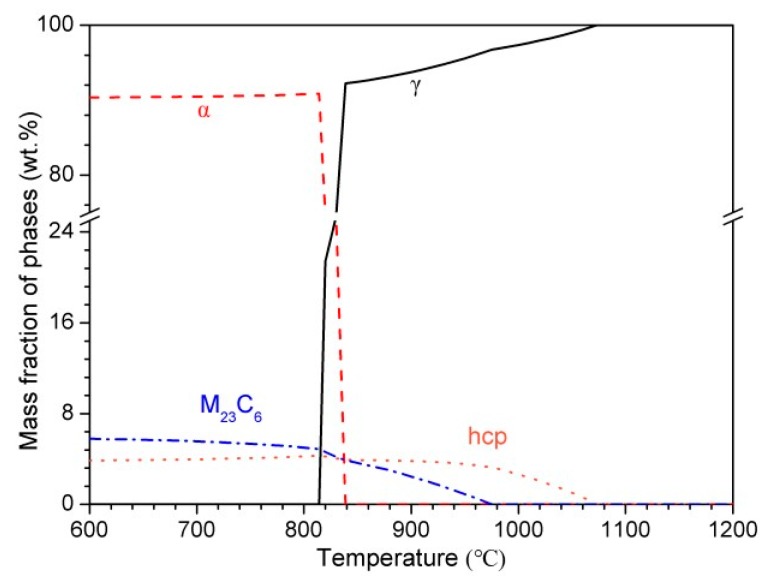
Variation of phase fractions with temperature in 30Cr15Mo1N.

**Figure 3:**
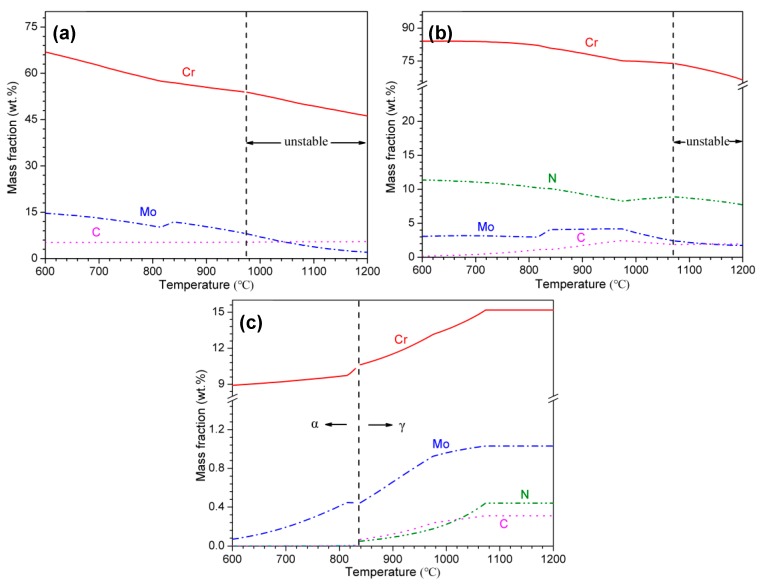
Variation in phase composition of: (**a**) M_23_C_6_; (**b**) M_2_N; and (**c**) matrix with temperature calculated by Thermo-Calc.

The variation of phase fractions with temperature in martensitic high nitrogen stainless steel 30Cr15Mo1N using Thermo-Calc software is presented in Figure 2. There existed two kinds of precipitates, i.e., M_23_C_6_ and hexagonal close-packed (hcp) phase. According to previous work by Mehtedi et al. [13] and Kaluba et al. [15], the hcp phase was preliminarily inferred to be Cr-rich M_2_N. The contents of M_23_C_6_ and M_2_N both decreased with the increasing of temperature, whereas the dissolution of M_23_C_6_ occurred ahead of M_2_N due to the higher diffusivity of carbon respect to nitrogen [13,15]. In contrast with traditional carbon alloyed MSSs, the existence of M_2_N with higher thermal stability in 30Cr15Mo1N enhanced the required austenitizing temperature in order to obtain uniform distribution of elements. In addition, Figure 3 shows the variation of phase composition with temperature. The Cr and Mo contents in M_23_C_6_ and M_2_N decreased, and the Cr, Mo, C and N contents in matrix increased with increasing austenitizing temperature.

### 3.2. Microstructure Characterizations

#### 3.2.1. X-ray Diffraction and Hardness Tests

**Figure 4:**
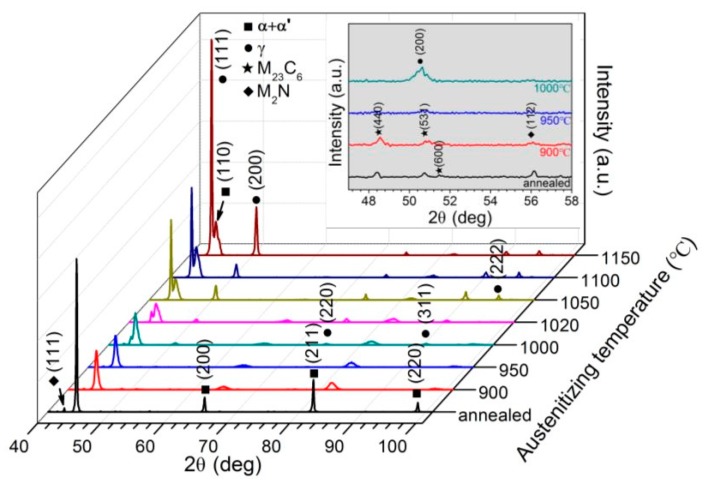
XRD patterns of 30Cr15Mo1N in annealing and austenitizing conditions.

**Figure 5:**
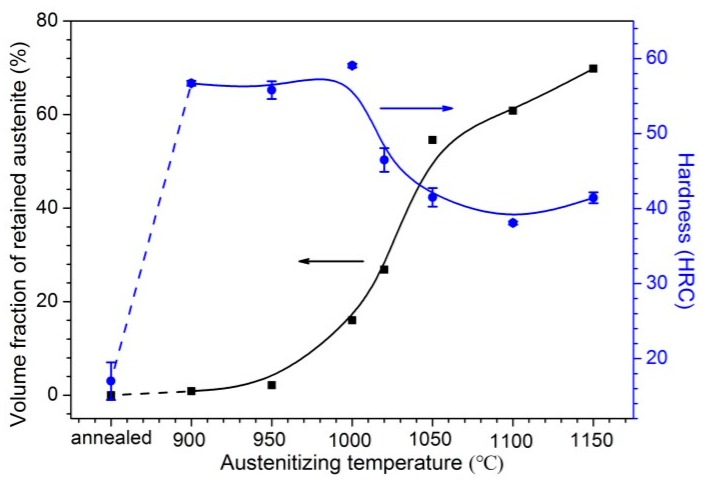
Variation of retained austenite content and hardness with austenitizing temperature.

Figure 4 plots the XRD patterns of 30Cr15Mo1N in annealing and austenitizing conditions. The diffraction peaks of body-centered cubic (bcc) phase were observed in all specimens. However, it is impossible to separate the ferrite (α) and martensite (α’) phases owing to the identical lattice constants and structure [20,22]. Thus, Rockwell hardness was measured using HRS-150D tester (Jvjing Precision Instrument Manufacturing Co., Ltd., Shanghai, China) with Rockwell C scale to identify these two phases, as shown in Figure 5. It is noted that the hardness value of annealed specimen was significantly lower than those of the austenitized ones. Therefore, the bcc phases detected by XRD spectra were identified to be ferrite in annealing condition and martensite in austenitizing condition [20]. With the increasing of austenitizing temperature, the peaks for γ phase were enhanced, indicating increased content of retained austenite. Due to the dissolution of M_23_C_6_ and M_2_N, the contents of carbon and nitrogen in matrix were raised with increasing austenitizing temperature, thereby depressing the martensitic transformation.

In addition, from the local amplification of XRD spectra from 47° to 58° in Figure 4, weak peaks for M_23_C_6_ and M_2_N were found in annealed and austenitized at 900 °C specimens. With increasing austenitizing temperature to 950 °C, the peaks for M_23_C_6_ and M_2_N weakened, and then disappeared above 1000 °C. However, the non-appearance of peaks for austenite or precipitates in XRD profiles meant the absence of these phases or the contents were lower than the detection limit [29].

#### 3.2.2. Microscopy Observation

**Figure 6:**
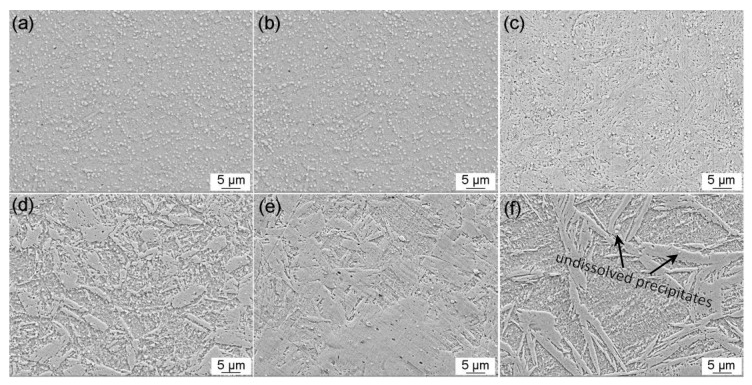
Morphologies of 30Cr15Mo1N austenitized at: (**a**) 900 °C; (**b**) 950 °C; (**c**) 1000 °C; (**d**) 1050 °C; (**e**) 1100 °C; and (**f**) 1150 °C.

The morphologies of 30Cr15Mo1N austenitized at 900 °C, 950 °C, 1000 °C, 1050 °C, 1100 °C and 1150 °C are illustrated in Figure 6. The microstructure consisted of scattered precipitate particles, i.e., Cr-rich M_23_C_6_ and M_2_N, distributing in lath martensite and retained austenite. As shown in Figure 6 and Table 2, the fraction of precipitates decreased significantly as the austenitizing temperature increased to 1000 °C. When the austenitizing temperature was above 1050 °C, just a few undissolved precipitates and non-metallic inclusions existed in the matrix. In addition, no δ-ferrite or coarse eutectic carbides were observed among all the specimens austenitized in the range of 900–1150 °C, which is consistent with the Thermo-Calc result (Figure 2).

**Figure 7:**
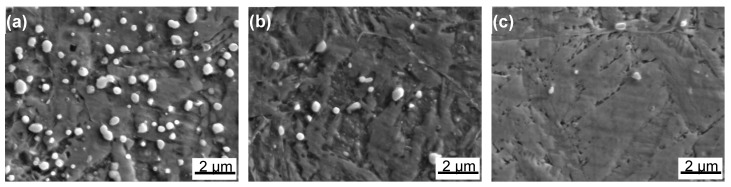

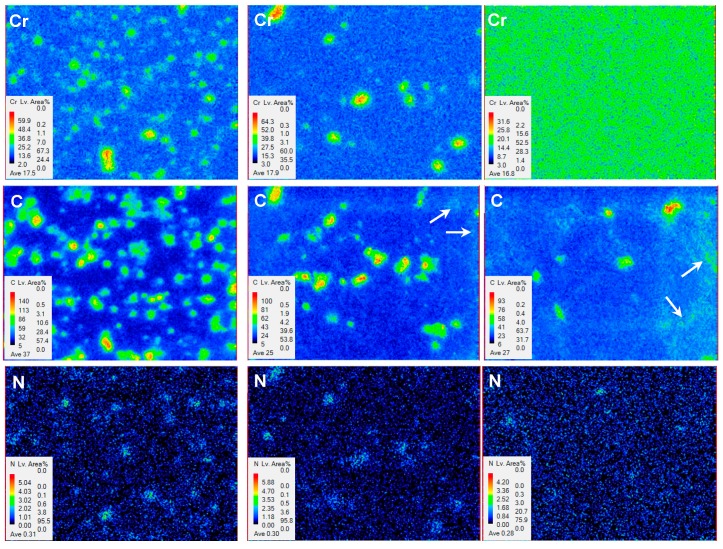
Element mapping of 30Cr15Mo1N austenitized at: (**a**) 900 °C; (**b**) 1000 °C; and (**c**) 1100 °C by EPMA.

The distribution of Cr, C and N via EPMA in Figure 7 shows that the precipitates were rich in Cr, C and N, and the quantity of N-rich particles was much lower than that of C-rich ones. The N-rich particles were finer and distributed more homogeneously than the C-rich ones. With the increasing of austenitizing temperature, the precipitates dissolved and the distribution of Cr, Mo, C and N became more homogeneous. Besides, the C content in lath martensite at 1000 °C and 1100 °C (indicated by arrows in Figure 7) was remarkably elevated. The extra dissolved C in the martensite would result in the increased internal martensite lattice stress, furthermore increasing the hardness, strength [19] and deteriorating the corrosion resistance [30].

**Figure 8:**
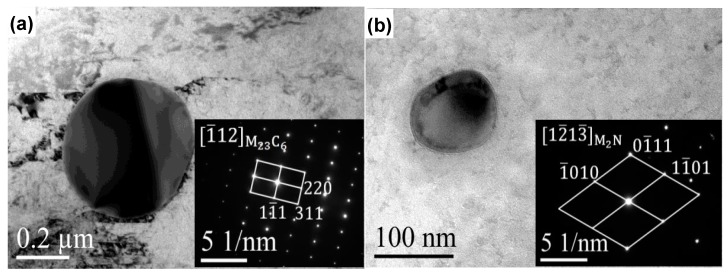

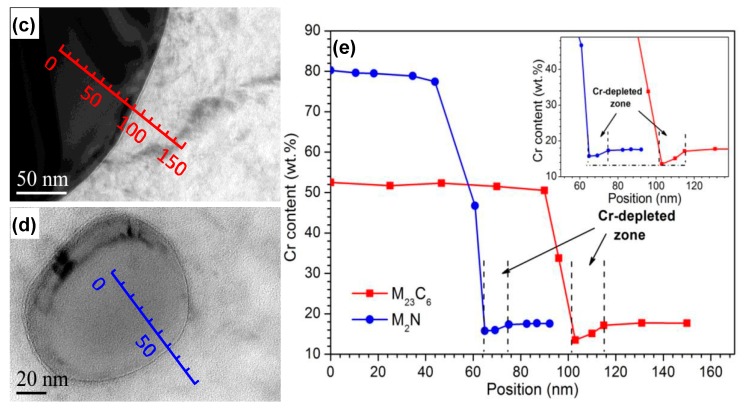
TEM results of 30Cr15Mo1N austenitized at 900 °C: (**a**,**b**) morphologies and SAED patterns; (**c**,**d**) high-magnification of precipitates in (**a**,**b**); and (**e**) Cr content profiles at the precipitate–matrix interface (The inset figure is the high magnification of Cr contents in Cr-depleted zones).

Figure 8 shows the TEM results of 30Cr15Mo1N austenitized at 900 °C. Spherical precipitates in the grain interior were observed, as shown in Figure 8a,b. Based on the selected area electron diffraction (SAED) patterns, the precipitates were identified as Cr-rich M_23_C_6_ and M_2_N, respectively. The M_2_N was finer than M_23_C_6_, which is consistent with the EPMA results (Figure 7). The high-magnification images and Cr concentration at the precipitate–matrix interface are illustrated in Figure 8c–e. The Cr content of M_2_N was obviously higher than that of M_23_C_6_, and the Cr-depleted zones were observed in the interface between M_23_C_6_/M_2_N and matrix. It is noteworthy that the widths of Cr-depleted zones around M_23_C_6_ and M_2_N were about 13.75 nm and 10.16 nm, respectively, and the minimum Cr contents were about 13.52 wt % and 15.78 wt %, respectively. Therefore, the Cr-depletion induced by coarse M_23_C_6_ was severer than M_2_N.

### 3.3. Electrochemical Measurement

**Figure 9:**
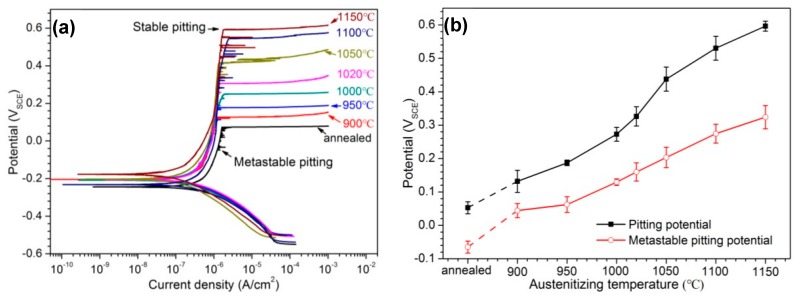
(**a**) Potentiodynamic polarization curves of 30Cr15Mo1N; and (**b**) change in metastable and stable pitting potentials with austenitizing temperature.

The potentiodynamic polarization curves of 30Cr15Mo1N in annealing and austenitizing conditions are presented in Figure 9a. There were obvious passive ranges where the current density remained almost stable. However, several current transients relating to the initiation and repassivation of metastable pits [20,31] can be observed in passive regions. The current density increased dramatically above the pitting potential, indicating the stable pitting corrosion occurred. As shown in Figure 9b, with the increasing of austenitizing temperature, both the metastable and stable pitting potentials increased, indicating the pit initiation became more difficult and the corrosion resistance was enhanced. In addition, the difference between metastable and stable pitting potentials also increased, which meant the repassivation tendency of metastable pitting became larger [32].

**Figure 10:**
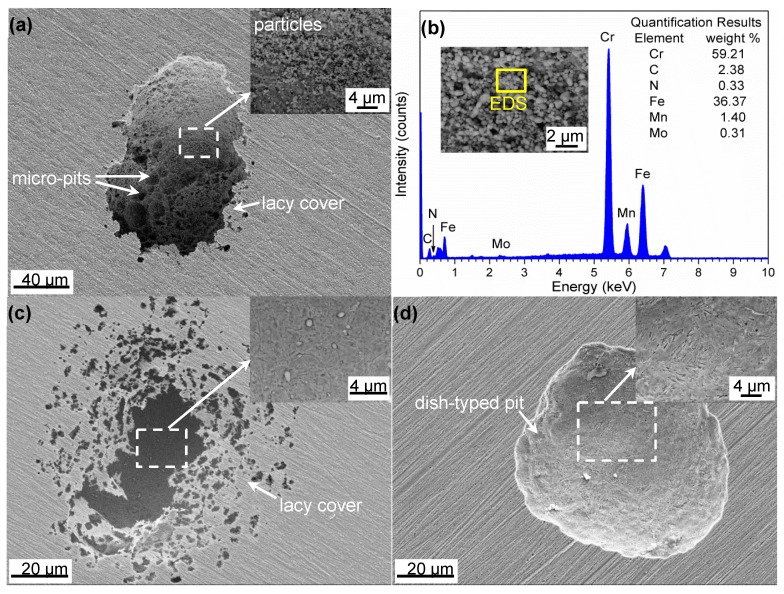
Morphologies of the largest corrosion pits in 30Cr15Mo1N austenitized at different temperatures after potentiodynamic polarization: (**a**) 900 °C; (**b**) EDS spectrum and high-magnification of particles in (**a**); (**c**) 1000 °C; and (**d**) 1100 °C.

The morphologies of the largest corrosion pits on 30Cr15Mo1N austenitized at 900 °C, 1000 °C and 1100 °C after potentiodynamic polarization are shown in Figure 10. The size and depth of pits decreased with the increasing of austenitizing temperature. As shown in Figure 10a,c, the pits in specimens austenitized at 900 °C and 1000 °C were presented as holes with collapsed lacy cover. The existence of these pits was related to passivation and undercutting near the pit mouth [31]. In addition, several micro-pits (indicated by arrows in Figure 10a) nucleated and grew at the bottom of primary pit in specimen austenitized at 900 °C. From the high magnification micrograph (image inserted into Figure 10a), there existed a mass of particles at the bottom of pit, which were confirmed to be rich in Cr according to the EDS spectrum (Figure 10b). The combination of chemical composition and high-magnification morphology indicated that these particles originated from the uncorroded precipitates in the matrix. When the austenitizing temperature increased to 1000 °C, just several particles existed at the pit bottom because of the dissolution of precipitates (Figure 10c). As to the specimen austenitized at 1100 °C, almost no particles existed in shallow dish-typed pit (Figure 10d), and the pit was significantly shallower than those austenitized at lower temperatures.

**Figure 11:**
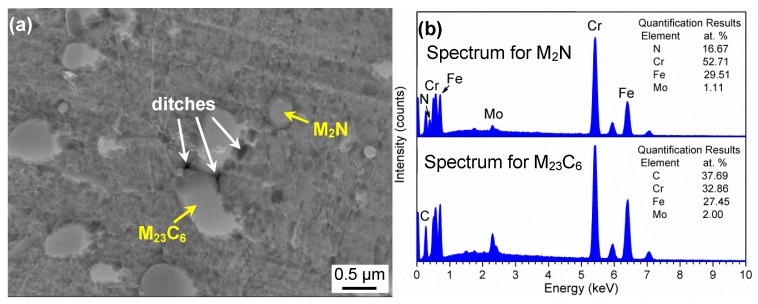
(**a**) Morphology of pit initiation sites; and (**b**) EDS spectra of precipitates in 30Cr15Mo1N austenitized at 900 °C.

To clearly present the metastable pit initiation sites, a specimen austenitized at 900 °C was mechanically abraded and polished. Then, it was observed using FE-SEM after potentiodynamically polarized to the metastable pitting potential. As shown in Figure 11a, there existed obvious ditches around large-sized precipitates, whereas no obvious ditches around small-sized precipitates were found. EDS spectra in Figure 11b indicated the large and small precipitates were Cr-rich M_23_C_6_ and M_2_N, respectively. Therefore, the ditches around M_23_C_6_ were confirmed to be the preferential metastable pit initiation sites, which might propagate to form stable pits.

### 3.4. XPS Results

**Figure 12:**
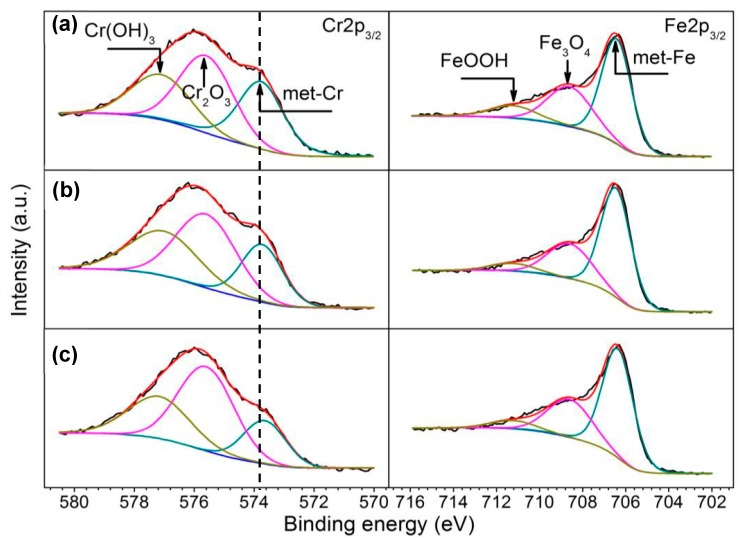
X-ray photoelectron spectra of Cr 2p_3/2_ and Fe 2p_3/2_ recorded from the passive films after sputtering for 10 s on 30Cr15Mo1N austenitized at (**a**) 900 °C; (**b**) 1000 °C; and (**c**) 1100 °C.

XPS analysis was performed to determine the effect of austenitizing temperature on the composition of passive films. Prior to the tests, the specimens were polarized at 0 mV_SCE_ (about 200 mV higher than OCP) for 1 h to promote the growth of passive films. Figure 12 shows Cr 2p_3/2_ and Fe 2p_3/2_ spectra of sputtering surface of passive film for 10 s. The Cr 2p_3/2_ spectra were split into Cr(OH)_3_ (576.9 eV), Cr_2_O_3_ (575.6 eV) and Cr metal (573.7 eV), and the Fe 2p_3/2_ spectra were divided into FeOOH (711.2 eV), Fe_3_O_4_ (708.6 eV) and Fe metal (706.5 eV). The binding energies of Cr metal slightly decreased with the increasing of austenitizing temperature, which was consistent with the dissolution of Cr-rich precipitates which had higher binding energies. With increasing austenitizing temperature, the peaks for Cr metal weakened, demonstrating the thickening of the passive film [33]. The components of passive films on 30Cr15Mo1N based on the fitting data (Figure 12) are listed in Table 3. The Cr_2_O_3_ content and ratio of Cr^3+^ to the sum of Fe^2+^ and Fe^3+^ increased with increasing austenitizing temperature. The variation in content of Cr_2_O_3_ and Cr^3+^ in the passive films demonstrates that Cr_2_O_3_ and Cr^3+^ were enriched by enhancing the austenitizing temperature.

**Figure 13:**
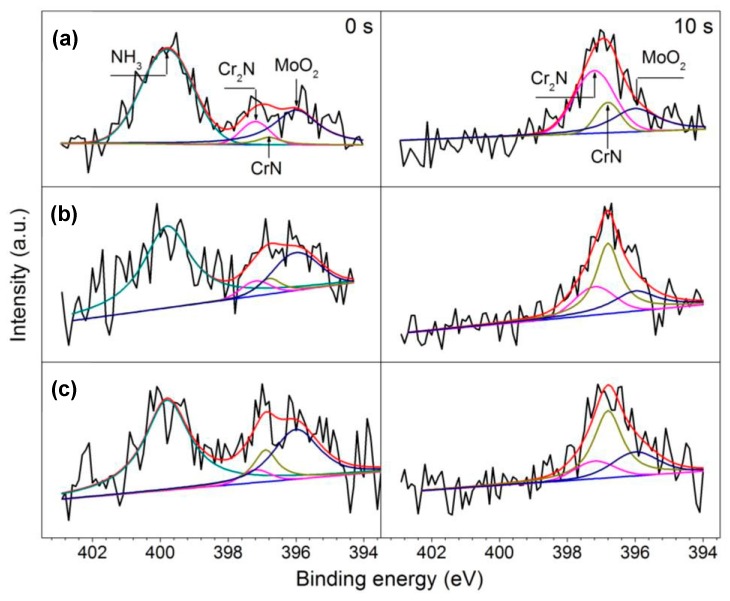
X-ray photoelectron spectra of N 1s and Mo 3p_3/2_ recorded from the outmost and sputtering surfaces of the passive films for 10 s on 30Cr15Mo1N austenitized at: (**a**) 900 °C; (**b**) 1000 °C; and (**c**) 1100 °C.

Figure 13 shows the X-ray photoelectron spectra of N 1s and Mo 3p_3/2_ recorded from the passive films after sputtering for 0 s and 10 s. On the outmost surface of passive films, peaks representing NH_3_ (399.8 eV), Cr_2_N (397.2 eV), CrN (396.8 eV) for N 1s and MoO_2_ (396.1 eV) for Mo 3p_3/2_ were detected. After sputtering for 10 s, the spectra exhibited peaks for Cr_2_N, CrN and MoO_2_, whereas peaks for NH_3_ disappeared. As the austenitizing temperature increased, the intensity of Cr_2_N in the passive films decreased while the intensity of CrN increased, which is associated with the dissolution of M_2_N precipitates at higher austenitizing temperature.

### 3.5. Immersion Tests

**Figure 14:**
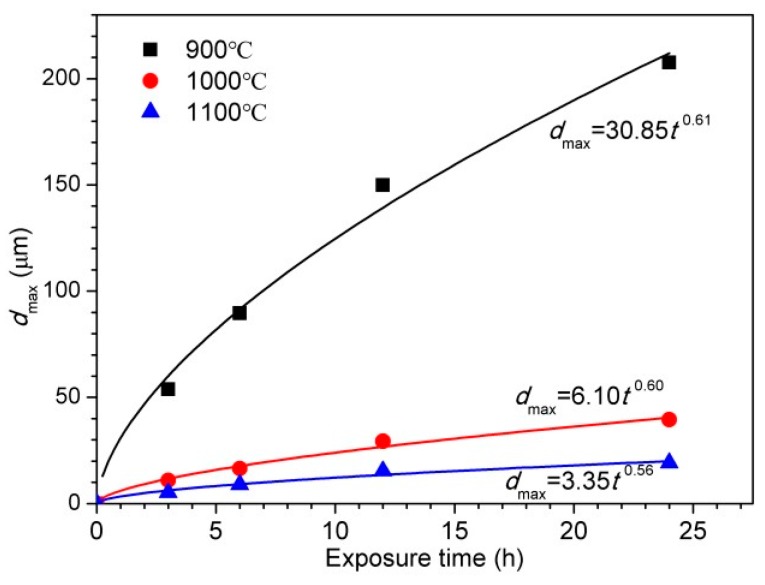
Evolution of maximum pit depth with immersion time for 30Cr15Mo1N austenitized at 900 °C, 1000 °C and 1100 °C.

The pitting corrosion and general corrosion occurred during immersion tests in 6 wt % FeCl_3_ at 50 ± 0.5 °C, thus both of them should be considered. The general corrosion rates for 30Cr15Mo1N austenitized at 900 °C, 1000 °C and 1100 °C are 23.53 mm/year, 4.10 mm/year and 1.33 mm/year, respectively. The specimen austenitized at 900 °C contained massive Cr-rich precipitates and the lowest chromium content in the matrix, thus having the highest general corrosion rate. With the increasing of austenitizing temperature, the chromium content in matrix increased, inducing the enhanced general corrosion resistance. The evolution of maximum pit depth considering general corrosion on specimens austenitized at 900 °C, 1000 °C and 1100 °C after being immersed for 3 h, 6 h, 12 h and 24 h is shown in Figure 14. It has been reported that the pit growth follows Equation (2) [34,35]:

*d*_max_ = *kt^n^* (2)

where *d*_max_ (μm) is the maximum pit depth, *k* (μm/h^−*n*^) and *n* are constants, and *t* (h) is the immersion time. It can be seen that the maximum pit depth increased with increasing immersion time, which followed the power law. With the increasing of austenitizing temperature, the maximum pit depth decreased significantly. The fitted parameters in Figure 14 showed that the values of *n* merely varied (approximately 0.5) and the values of *k* decreased significantly with increasing austenitizing temperature, revealing a lower pit growth rate for specimen austenitized at higher temperature.

## 4. Discussion

Based on the above results, the reason for preferential metastable pit initiation around coarse M_23_C_6_, and the relationship between microstructure and corrosion resistance of martensitic high nitrogen stainless steel 30Cr15Mo1N at different austenitizing temperatures by analyzing pit initiation, passive film and pit growth kinetics will be discussed in detail.

### 4.1. Explanation of Preferential Metastable Pit Initiation Sites

The martensitic high nitrogen stainless steel 30Cr15Mo1N contained Cr-rich M_23_C_6_ and M_2_N, which were different in distribution, size, volume fraction, chemical composition and Cr-depleted zone (Figure 2, Figure 7 and Figure 8). Initially, the M_2_N precipitated during annealing process was more coherent than M_23_C_6_ in nitrogen alloyed MSSs [18,36], resulting in the smaller size and more homogeneous distribution of M_2_N than M_23_C_6_. During the austenitization process, M_2_N and M_23_C_6_ partially dissolved, and the nitrogen-induced short range atomic ordering and the strong Cr–N bond prevented the chromium clustering in 30Cr15Mo1N [16,36]. Then in quenching process, the martensite inherited from the austenite possessed a homogeneous element distribution. The content of M_2_N was lower than M_23_C_6_ because the atomic fraction of N in M_2_N was higher than C in M_23_C_6_. Besides, the addition of nitrogen suppressed the precipitation of coarse eutectic carbides, which was due to higher austenite stability upon quenching [36] and higher binging energy of Cr–N than that of Cr–C [16]. Similar results have been obtained in Cr15Mo1 [16] and SUS440A [17] with different nitrogen contents.

It is well accepted that pitting corrosion initiates from breakdown of passive film or chemical/physical heterogeneity, such as inclusions and precipitates [37,38]. The detrimental effect of Cr-rich precipitates [39,40,41,42], such as Cr_23_C_6_, Cr_2_N and σ, on corrosion resistance due to Cr-depletion has been widely reported. In the present study, Cr-rich M_23_C_6_ and M_2_N were generated in the annealing process, resulting in the emerging of Cr-depleted zones in the vicinity of precipitate/matrix interface simultaneously. During the austenitization process, M_23_C_6_ and M_2_N partially dissolved into the matrix. As shown in Figure 8, coarse M_23_C_6_ induced wider and severer Cr-depleted zone than M_2_N, which resulted in the preferential initiation of metastable pits around M_23_C_6_ (Figure 11). On the one hand, the lower Cr content in Cr-depleted zone induced poorer stability of passive film, which was vulnerable to become initiation sites for pitting corrosion. On the other hand, the chemical composition diversity between matrix and Cr-depleted zone led to the formation of micro-cell with narrow anodic zone [36], thus increasing the pitting corrosion sensitivity.

### 4.2. Influence of Austenitizing Temperature on Pit Initiation

As discussed in Section 4.1, metastable pits initiated around Cr-rich precipitates, preferentially the coarse M_23_C_6_ with severer Cr-depletion. The content of precipitates decreased with increasing austenitizing temperature (Figure 6), which meant the number of pit initiation sites was reduced. Choi et al. [21] and Park et al. [22] reported that the pitting potential of MSSs was enhanced as the austenitizing temperature increased, which was attributed to the decrease of carbides. In the present research, the metastable and stable pitting potentials increased with increasing austenitizing temperature, revealing less susceptible metastable pit initiation and higher corrosion resistance. Moreover, the difference between metastable and stable pitting potentials at low austenitizing temperature was small, which indicates that the transition of metastable pits to stable pits occurred soon after the initiation of metastable pits and the repassivation ability was weak. With the increasing of austenitizing temperature, the difference between metastable and stable pitting potentials increased simultaneously, demonstrating that although the initiation of metastable pits occurred at relatively low potential, but the metastable pits could be transformed into stable pits only at much higher potential [32].

Additionally, it is generally believed that high temperature promotes the atomic diffusion. As shown in Figure 3, with increasing austenitizing temperature, the decreased Cr and Mo contents in M_23_C_6_ and M_2_N and the increased Cr, Mo, C and N contents in matrix resulted in higher degree of composition homogeneity in 30Cr15Mo1N. The Cr atoms diffused from both precipitates and matrix to the Cr-depleted zones where the Cr content was the lowest. The Cr content of Cr-depleted zones was compensated to a higher extent when austenitized at higher temperature due to more rapid diffusion of Cr atoms, thus also delaying the initiation of pitting corrosion.

**Figure 15:**
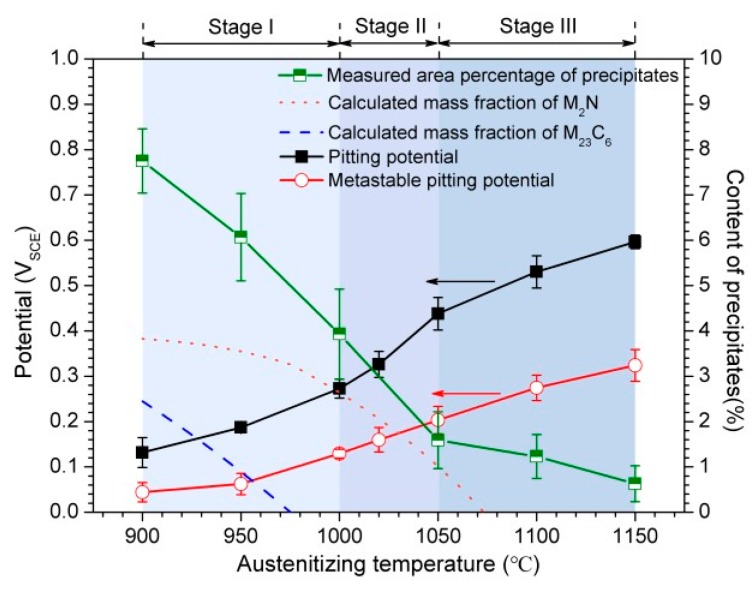
Correlation between content of precipitates and pitting potentials of 30Cr15Mo1N austenitized at different temperatures.

The correlation between content of precipitates and pitting potentials of 30Cr15Mo1N austenitized at different temperatures is shown in Figure 15. The variation of pitting potentials could be divided into three stages. At the temperature below 1000 °C (Stage I), although M_23_C_6_ dissolved gradually, the existence of massive M_23_C_6_ induced the relatively low and slow increase of the metastable and stable pitting potentials with the increasing of temperature. The M_23_C_6_ dissolved completely around 975 °C, and the content of M_23_C_6_ decreased obviously when the temperature reached 1000 °C (Figure 7). Accordingly, a sharp rise of metastable pitting potential (from 950 to 1000 °C) was observed in Figure 15. Since then, the metastable pitting potential increased stably with the increment of austenitizing temperature. When the temperature increased to 1000 °C, the content of precipitates decreased obviously (Figure 6), and the precipitates almost disappeared at temperatures higher than 1050 °C. The dissolution of M_2_N could be obtained from the Thermo-Calc results from 1000 to 1075 °C. In accordance with this, the increasing rate of stable pitting potential accelerated, particularly in the range of 1000 °C to 1050 °C (Stage II). After that, only a few undissolved precipitates existed, and the decrease rate of precipitate content reduced when the temperature was higher than 1050 °C (Stage III), which was consistent with the slow increasing rate of stable pitting potential. Therefore, the determining factor of the three stages for pitting potential could be dissolution of M_23_C_6_ (below 1000 °C), dissolution of M_2_N (from 1000 to 1050 °C) and existence of a few undissolved precipitates and non-metallic inclusions (above 1050 °C), respectively.

### 4.3. Influence of Austenitizing Temperature on Passive Film

With increasing austenitizing temperature, the Cr-rich precipitates dissolved and the Cr content in matrix was enhanced, promoting the thickening of the passive films and enrichment of Cr_2_O_3_ and Cr^3+^ (Figure 12 and Table 3). Besides, the nitrogen content in matrix increased as austenitizing temperature rose, which is beneficial to the enrichment of Cr_2_O_3_ in the passive films [10,33]. Additionally, the interaction of nitrogen and molybdenum promoted the deprotonation of Cr-hydroxide by inducing O–H bond stretching, which also promoted the enrichment of Cr_2_O_3_ [33,43]. On the other hand, compared with Cr(OH)_3_, Cr_2_O_3_ had higher thermodynamic stability [44] and lower point defect concentration [45], thus enhancing the stability of passive film and hindering pit initiation [37].

Based on the deconvolution of N spectra (Figure 13), two kinds of nitrides, i.e., CrN and Cr_2_N, were detected. The Cr_2_N corresponded to the undissolved Cr-rich M_2_N, which had high Volta potential [46] and hardly corroded in the corrosion process, as shown in Figure 10. Therefore, it could not consume protons in pits and had no positive effect on corrosion resistance of 30Cr15Mo1N. However, nitrogen in solid solution was anodically segregated to be CrN in the passive film during pitting corrosion process [7]. The CrN could consume protons to form ammonium ions by Equation (3) [47]:

2CrN + 3H_2_O → Cr_2_O_3_ + 2NH_3_ (3)

consequently hindering the self-catalytic process and promoting the repassivation of pits [33,48]. With increasing austenitizing temperature, the dissolution of M_2_N induced the decrease of Cr_2_N intensity in passive film. Thereafter, the enhanced content of nitrogen in solid solution promoted the increasing of CrN content in the passive film, which could consume more protons and promote the repassivation of incipient pits.

### 4.4. Effect of Austenitizing Temperature on Pit Growth Kinetics

After the initiation of metastable pit, it might propagate to form a stable pit and grow self-catalytically. The pit growth followed the power law (Figure 14), and its rate was determined by the values of *n* and *k*. In the present work, the values of *n* were approximately 0.5 independent of the austenitizing temperature. Cavanaugh et al. [48] reported that the value of *n* equaled to 0.5 when the pit growth was under ohmic or diffusion control. The values of *k* considerably decreased with the increasing of austenitizing temperature, indicating lower pit growth rate, which was in agreement with the pit morphologies after potentiodynamic polarization (Figure 10). Nitrogen in solid solution could be released to the pits in the form of NH_3_ and/or NH_4_^+^ by Equations (4) and (5), respectively, in the pitting corrosion process [33,43]:

[N] + 3H^+^ + 3e^−^ → NH_3_ (4)

[N] + 4H^+^ + 3e^−^ → NH_4_^+^ (5)

and protons in pits were partially consumed. The higher austenitizing temperature enhanced the content of nitrogen in solid solution, promoting the repassivation ability of pits [33], which contributed to lower pit growth rate and shallower pits.

**Figure 16:**
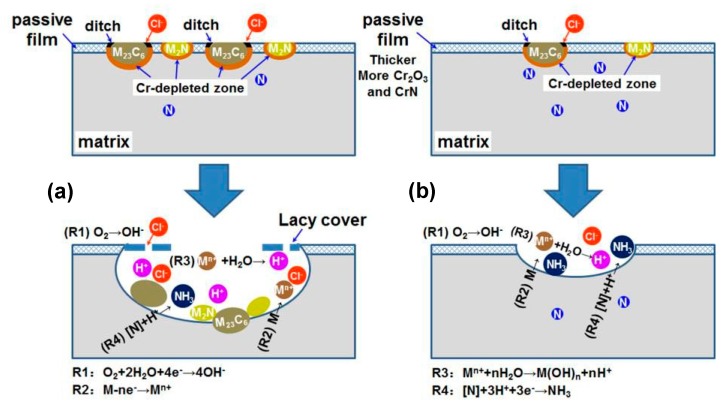
Schematic models for corrosion mechanism of 30Cr15Mo1N austenitized at: (**a**) lower; and (**b**) higher temperatures.

Based on the analysis of microstructure, electrochemical behavior, passive film, pit initiation and propagation, the relationship between microstructure and corrosion resistance of 30Cr15Mo1N at different austenitizing temperatures is schematically presented in Figure 16. When the specimens were polarized to high potential in chloride solution, metastable pits initiated preferentially around coarse M_23_C_6_ with severer Cr-depletion. With fewer pit initiation sites and more protective passive film (thicker and enriched in Cr_2_O_3_, Cr^3+^ and CrN), the specimen austenitized at higher temperature corroded less severely than that austenitized at lower temperature. With the proceeding of pitting corrosion, pits grew by self-catalytic mechanism. The uncorroded Cr-rich M_23_C_6_ and M_2_N accumulated at the bottom of pits in specimen austenitized at lower temperature. The higher austenitizing temperature induced enhanced content of nitrogen in solid solution in the matrix, which promoted the repassivation process and retarded the growth of pits, resulting in shallower pits.

## 5. Conclusions

In the present work, the relationship between microstructure and corrosion behavior of martensitic high nitrogen stainless steel 30Cr15Mo1N at different austenitizing temperatures was investigated using microscopy observation, electrochemical measurement, passive film analysis and immersion testing. The main conclusions could be obtained as follows:

(1) With increasing austenitizing temperature, the fraction of precipitates decreased and retained austenite increased, resulting in more homogeneous distribution and higher contents of Cr, Mo, C and N in the matrix. The precipitates were identified as Cr-rich M_23_C_6_ and M_2_N, and M_2_N was finer and distributed more homogeneously than M_23_C_6_. The Cr-depleted zone around M_23_C_6_ was wider and its minimum Cr concentration was lower than M_2_N.
(2) The metastable pits initiated preferentially around coarse M_23_C_6_ which induced severer Cr-depletion, and the pit growth followed the power law. The dissolution of M_23_C_6_ and M_2_N at higher austenitizing temperature reduced the pit initiation sites. As austenitizing temperature increased, the metastable and stable pitting potentials increased and the pit growth rate decreased, revealing less susceptible metastable pit initiation, larger repassivation tendency and higher corrosion resistance. The determining factor of pitting potential could be divided into three stages: dissolution of M_23_C_6_ (below 1000 °C), dissolution of M_2_N (from 1000 to 1050 °C) and existence of a few undissolved precipitates and non-metallic inclusions (above 1050 °C).
(3) The increasing of austenitizing temperature promoted the thickening of passive film; enrichment of Cr_2_O_3_, Cr^3+^ and CrN; and higher nitrogen content in solid solution, thereby enhancing the stability of passive film and repassivation ability of pits.

## Figures and Tables

**Table 1:** Chemical composition of martensitic high nitrogen stainless steel 30Cr15Mo1N (wt %).

C	Cr	Mo	N	Mn	Si	Ni	S	P	O
0.31	15.17	1.03	0.44	0.44	0.52	0.07	0.002	0.011	0.0015

**Table 2:** Area percentage of precipitates in 30Cr15Mo1N austenitized at different temperatures.

Temperature (°C)	900	950	1000	1050	1100	1150
Area percentage of precipitates (%)	7.75 ± 0.71	6.07 ± 0.96	3.94 ± 0.99	1.59 ± 0.63	1.23 ± 0.48	0.63 ± 0.40

**Table 3:** Component of passive films on 30Cr15Mo1N austenitized at 900 °C, 1000 °C and 1100 °C.

Specimens	Component of Passive Films (at %)				Cr^3+^/(Fe^2+^ + Fe^3+^)
	Fe_3_O_4_	FeOOH	Cr_2_O_3_	Cr(OH)_3_	
900 °C	12.51	4.68	6.97	4.52	0.24
1000 °C	11.39	2.49	8.47	6.34	0.29
1100 °C	11.92	2.56	9.04	5.33	0.31
